# Transfer of fibroblast sheets cultured on thermoresponsive dishes with membranes

**DOI:** 10.1007/s10856-016-5718-1

**Published:** 2016-05-06

**Authors:** Marek Kawecki, Małgorzata Kraut, Agnieszka Klama-Baryła, Wojciech Łabuś, Diana Kitala, Mariusz Nowak, Justyna Glik, Aleksander L. Sieroń, Alicja Utrata-Wesołek, Barbara Trzebicka, Andrzej Dworak, Dawid Szweda

**Affiliations:** Dr Stanislaw Sakiel Centre for Burns Treatment, Jana Pawła II 2, 41-100 Siemianowice Śląskie, Poland; Faculty of Health Sciences, University of Bielsko-Biala, Willowa 2, 43-309 Bielsko-Biała, Poland; Department of Molecular Biology and Genetics, Medical University of Silesia, Medyków 18, 40-752 Katowice, Poland; Centre of Polymer and Carbon Materials, Polish Academy of Sciences, M. Curie-Sklodowskiej 34, 41-819 Zabrze, Poland

## Abstract

In cell or tissue engineering, it is essential to develop a support for cell-to-cell adhesion, which leads to the generation of cell sheets connected by extracellular matrix. Such supports must be hydrophobic and should result in a detachable cell sheet. A thermoresponsive support that enables the cultured cell sheet to detach using only a change in temperature could be an interesting alternative in regenerative medicine. The aim of this study was to evaluate plates covered with thermoresponsive polymers as supports for the formation of fibroblast sheets and to develop a damage-free procedure for cell sheet transfer with the use of membranes as transfer tools. Human skin fibroblasts were seeded on supports coated with a thermoresponsive polymer: commercial UpCell™ dishes (NUNC™) coated with thermoresponsive poly(N-isopropylacrylamide) (PNIPAM) and dishes coated with thermoresponsive poly(tri(ethylene glycol) monoethyl ether methacrylate) (P(TEGMA-EE)). Confluent fibroblast sheets were effectively cultured and harvested from both commercial PNIPAM-coated dishes and laboratory P(TEGMA-EE)-coated dishes. To transfer a detached cell sheet, two membranes, Immobilon-P^®^ and SUPRATHEL^®^, were examined. The use of SUPRATHEL for relocating the cell sheets opens a new possibility for the clinical treatment of wounds. This study established the background for implementing thermoresponsive supports for transplanting in vitro cultured fibroblasts.

## Introduction

The outer layer of the skin, the epidermis, is composed mostly of epithelial cells (keratinocytes), pigment cells (melanocytes), cells responsible for immune reactions (Langerhans cells) and nervous system cells (Merkel’s cells), whereas fibroblasts are connective tissue cells that inhabit the dermis. Connective tissue, the main component of the dermis, is composed mostly of collagen and elastin fibers [1]. Skin cells can proliferate ex vivo in cell culture under appropriate conditions. Without the ability to adhere to the surface of a culture flask, these types of cells cannot proliferate. Therefore, the cells are cultured in an appropriate medium to ensure cellular adhesion to the bottom of the flask [2], which is often made of modified polystyrene tissue culture polystyrene (TCPS) [3]. Under in vitro conditions, a homogeneous sheet of cells connected by extracellular matrix (ECM) can be obtained. After skin cell sheet formation, the transfer to a wound can be problematic [4]. The skin cells must be separated from the support [5]. There are two basic methods that are used for cell separation, mechanical and enzymatic separation. Mechanical separation is based on cell scraping with special scrapers. However, it damages the cells. Cell separation can also be performed with the use of proteases (e.g., dispase). This method is commonly used and is less invasive. Proteases cause the enzymatic degradation of the ECM, which ultimately leads to cell separation [6]. The layer of cells is disintegrated when full confluence has not been reached or the connections between cells are weak. The enzymes can also destroy (digest) cell surface receptors that are needed for cell re-adhesion to the new surfaces, e.g., wounds [7, 8]. Enzymatic degradation may cause death of some cells, especially in the case of prolonged exposure to the enzymes [3, 9, 10].

To avoid cell sheet disintegration, cells, with the support still intact, can be placed onto a wound; thus, the cell separation process can be avoided. In such situations, the support must be surgically removed later, which affects the patient’s organism and is often painful. An exception to surgical removal is the situation where the support is biodegradable in vivo after implantation [4]. Despite the many advantages of biodegradable supports [4, 11], previous experiences have shown some limitations [12]. Most of the biodegradable supports are made of either lactide or glycolide polymers, and the degradation products of these materials are not neutral for the patient, even if they are non-toxic [13]. The most common complication is the strong acidification of the implant area and the induction of a nonspecific inflammatory response. Additionally, the grafting of supports along with the cell sheets causes difficulties in the diffusion of nutritional elements into the implant and in the removal of metabolites [4]. Therefore, cells will only proliferate on the periphery and will die on the internal parts of the implant. Another possibility to avoid cell sheet disintegration is the formation of a keratinocyte multilayer on murine fibroblasts grown on TCPS [14]. The keratinocyte multilayer was detached from the culture support during the enzymatic harvesting of fibroblasts [15, 16]. The most important disadvantage of this method is the contamination of keratinocyte multilayers with murine fibroblasts.

**Fig. 1:**
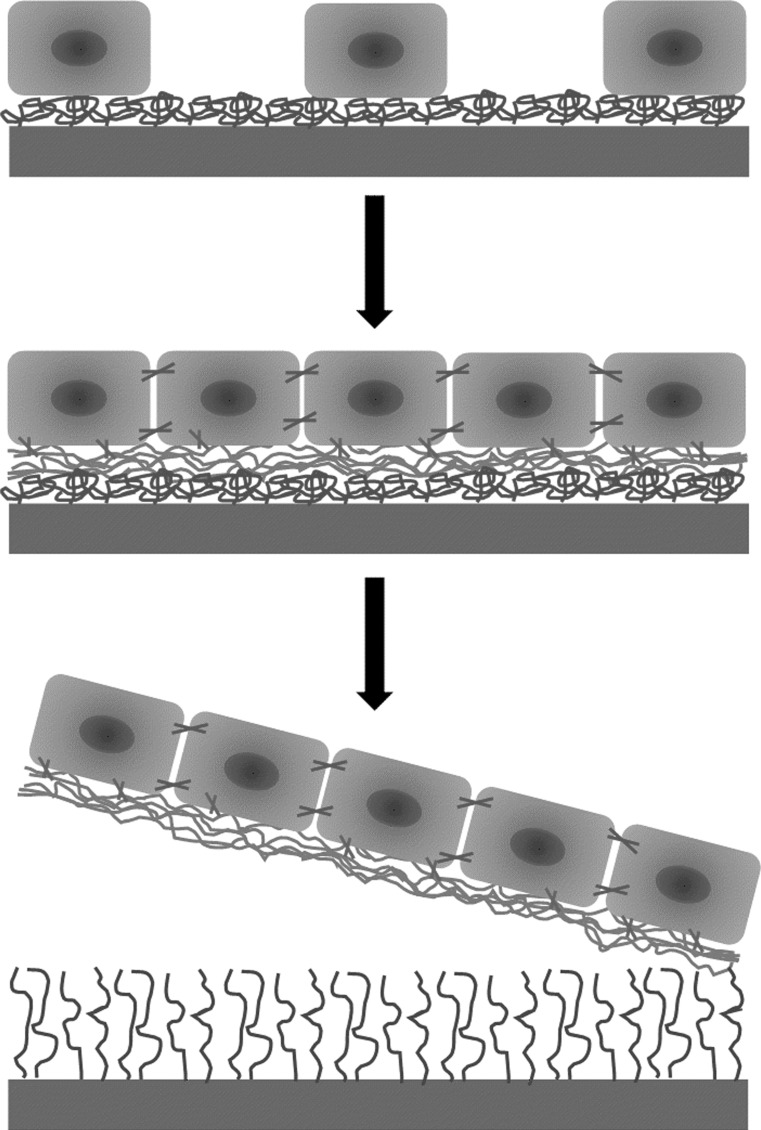
Separation of the cell sheet from the thermoresponsive support due to temperature changes

All these efforts indicate that there is a need for further research to establish a new methodology for the preparation of intact cell layers with possible applications in tissue engineering. The use of thermoresponsive polymers (TRPs) to develop supports with thermoresponsive properties is an alternative way to obtain suitable cell culture dishes for harvesting cell sheets [17]. A change in support hydrophilicity, which is induced by a change in environmental temperature, causes spontaneous cell sheet detachment from the support. In this method, the use of enzymes is avoided. This concept is depicted in Fig. 1, and it has been described in detail previously [18].

Dermal fibroblasts facilitate wound closure, affect the deposition of certain components of the epidermis [19] and support the adhesion and proliferation of keratinocytes [20]. In vitro-cultured keratinocyte and fibroblast sheet grafts have a less aggravating effect on the patient than autologous split-thickness skin grafts obtained from healthy, unaffected skin of the patient.

The aim of this study was to explore the possibility of the use of Immobilon-P^®^ and SUPRATHEL^®^ membranes to relocate/transfer cell sheets cultured on dishes coated with TRPs and detached by temperature stimulus to the target surface. Commercial NUNC UpCell dishes coated with poly(N-isopropylacrylamide) (PNIPAM) and dishes coated with poly(tri(ethylene glycol) monoethyl ether methacrylate) (P(TEGMA-EE)) prepared by the Centre of Polymer and Carbon Materials of the Polish Academy of Science (CMPW PAN) were used as supports for the culture of sheet-formed fibroblasts.

## Materials and methods

### Materials

UpCell™ dishes (manufactured by NUNC™) were purchased from Thermo Scientific. P(TEGMA-EE) dishes were prepared as described previously [21]. Immobilon-P^®^ membranes were purchased from Millipore Merck, and SUPRATHEL^®^ membranes were purchased from Medical BVBA. Lines of skin fibroblasts (not used for patient treatment) were obtained from split-thickness skin fragments that were harvested from patients hospitalized at the Centre for Burns Treatment and were used in the experiments.

TCPS 24-well plates, petri dishes (TPP AG), Dulbecco’s Modified Eagle Medium Advanced Therapy Medicinal Product (DMEM–ATMP), ready high glucose (4.5 g/L; +10 % Fetal Bovine Serum Advanced Therapy Medicinal Product (FBS–ATMP) +1 % l-Glutamine; PAA Laboratories GmbH) and trypsin in EDTA–ATMP (PAA Laboratories GmbH) were used as received. Dispase II (Gibco^®^) was used as received.

### Cell sheet culture

First, skin cells were separated from a portion of the split-thickness skin by placing it in a 2.4 U/mL dispase II solution to separate the epidermis from the dermis. The process was performed under specific conditions, including 37 °C, 5 % CO_2_ and 95 % humidity, for approximately 60 min. A suspension of single cells (from the dermis) was obtained using a 0.05 % trypsin–EDTA–ATMP solution. The primary culture of fibroblasts was performed in standard TCPS culture flasks with DMEM–ATMP supplemented with 10 % FBS–ATMP. When 80 % confluence was achieved, the fibroblasts were detached with the use of 0.05 % trypsin–EDTA–ATMP and seeded onto the polymer-coated dishes:

- UpCell dishes (manufactured by NUNC) coated with thermoresponsive PNIPAM,
- dishes coated with thermoresponsive P(TEGMA-EE) [21],
- TCPS dishes.

Before cell sheet culture, the polymer-coated dishes were conditioned: UpCell dishes in accordance with the manufacturer’s guidelines and dishes prepared by us according to the procedure described previously [21].

On both types of pre-incubated polymer coated-dishes, 250,000 fibroblasts were seeded per 1 cm^2^. The same amount of cells was seeded onto the control TCPS dishes. The cells were then cultured in DMEM–ATMP supplemented with 10 % FBS–ATMP (37 °C, 5 % CO_2_, 95 % humidity) for 24 h. To perform statistical analysis of cells experiment were repeated five times.

### Cell sheet detachment, transfer and re-adhesion

An attempt to separate the cell sheets from the UpCell dishes was performed in accordance with the manufacturer’s guidelines at 20 °C, whereas for the dishes coated with P(TEGMA-EE), this was performed at 17.5 °C. After 24 h (full cell coverage of culture dishes), the culture medium was replaced with a new one of lowered temperature (20 or 17.5 °C, respectively). The temperature-induced detachment of cell sheets from UpCell, P(TEGMA-EE) and TCPS (for comparison) dishes was observed using an inverted phase contrast microscope (Nikon Eclipse).

Next, an experiment to relocate/transfer a full-sized sheet of fibroblasts onto another dish was performed using a pipette (after dispase II detachment of the cell sheets) or special membranes: the Immobilon-P^®^ and SUPRATHEL^®^ (after cooling the cell sheet culture to 20 or 17.5 °C). Cell sheets obtained after 24 h of culture on UpCell, P(TEGMA-EE) and TCPS dishes were covered with the membranes. Forty minutes after lowering the temperature to 20 °C for UpCell and TCPS or to 17.5 °C for P(TEGMA-EE), the membranes with cells were transferred to new TCPS dishes. As a control, cells were detached from UpCell, P(TEGMA-EE) and TCPS dishes by dispase II treatment (2.4 U/mL, 37 °C) [6] and transferred using a pipette to new culture dishes (TCPS). The detachment yields were calculated using the following equation:

$${\text{detachment yield}} = \frac{{{\text{I}} - {\text{F}}}}{\text{I}} \times 1 0 0\%$$

where I is the number of cells after 24 h of culture, F is the number of cells left on the UpCell, P(TEGMA-EE) and TCPS dishes after detachment/transfer (remaining cells were detached with 0.05 % trypsin–EDTA–ATMP and counted using a Bio-Rad Cell Counter TC10).

Transferred cells (by pipette or membranes) were cultured on TCPS in DMEM–ATMP supplemented with 10 % FBS–ATMP (temperature 37 °C, 5 % CO_2_, 95 % humidity) for 24 h. Then, the membrane (if used) was removed, and the TCPS dishes with re-adhered cells were washed with fresh culture medium to remove the non-adhered cells. The re-adhesion yields were calculated from the equation:

$${\text{re-adhesion}}\,{\text{yield}} = \frac{{{\text{I}} - {\text{F}} - {\text{N}}}}{{{\text{I}} - {\text{F}}}} \times 100\%$$

where I is the number of cells after 24 h of culture, F is the number of cells left on the UpCell, P(TEGMA-EE) and TCPS dishes after detachment/transfer (remaining cells were detached with 0.05 % trypsin–EDTA–ATMP and counted using a Bio-Rad Cell Counter TC10), N is the number of cells that did not re-adhere (counted using a Bio-Rad Cell Counter TC10).

Re-adhered cells were observed using an inverted phase contrast microscope (Nikon Eclipse).

### Cell viability

The viability of the cells before transfer and cells transferred with membrane, re-adhered and after 24 h of culture detached with 0.05 % trypsin–EDTA–ATMP solution was measured using the Tali^®^ Viability Kit—Dead Green on a Tali^®^ Image Cytometer (Life Technologies™).

### Genotoxicity assay

The genotoxicity test was performed using dedicated Comet Assay Reagent Kit (Trevigen, USA), according to the manufacturer’s protocol. Common descriptor of DNA damage for alkaline comet assays is the tail moment olive, combining the amount of DNA in tail with distance of migration.

### Histological analysis

The harvested cell sheets were fixed with 3.7 % formaldehyde. The fixed specimens were embedded into paraffin and sliced into 4–5 μm-thick sections. Hematoxylin and eosin staining was performed by conventional methods.

### Statistical analysis

For statistical analysis STATISTICA 10 was used. The normality was tested with the Shapiro–Wilk test. For comparing more than two groups of independent samples The Kruskal–Wallis test was used. Significance level was set to 0.05 (5 %).

## Results

**Fig. 2:**
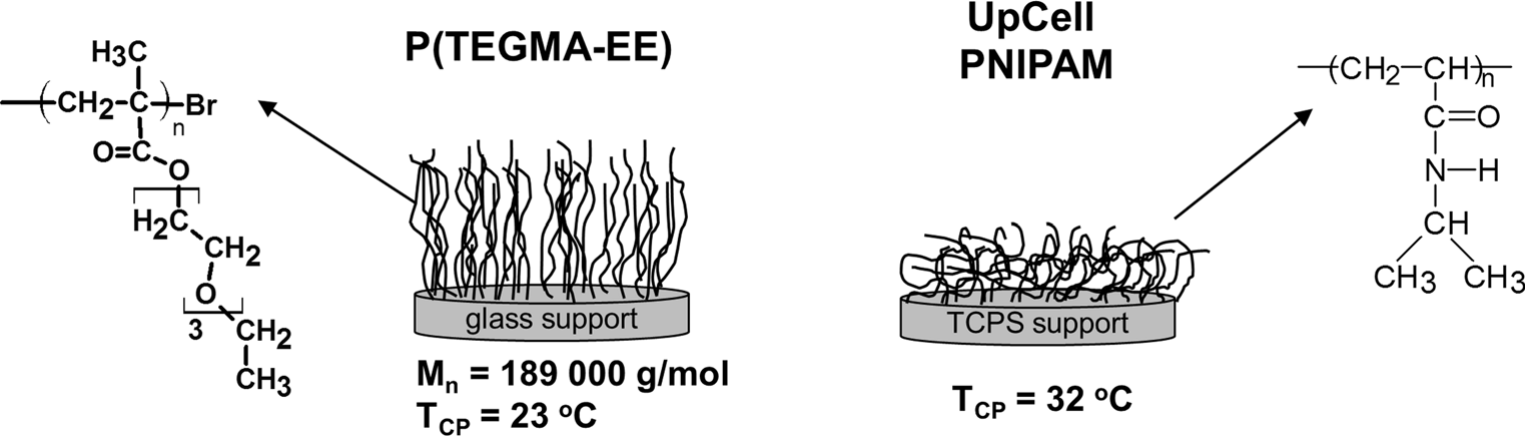
The properties of the PNIPAM and P(TEGMA-EE) dishes used for fibroblast culture

Cell culture and detachment were performed on the thermoresponsive PNIPAM (UpCell) and P(TEGMA-EE) surfaces (Fig. 2). The synthesis and properties of the P(TEGMA-EE) dishes were described previously [21]. The biocompatibility of both polymers in the culture solution was studied in a separate experiment, which indicated no toxicity of the polymers to the fibroblasts. The date on detachment, re-adhesion, transfer yields and viability of cells cultured on the UpCell and P(TEGMA-EE)-coated dishes are given in Table 1.

**Table 1:** The detachment, re-adhesion, transfer yields and viability of cells cultured on the UpCell and P(TEGMA-EE)-coated dishes

	UpCell dishes		P(TEGMA-EE)-coated dishes		Statistical differences
	Temperature stimulus/suprathel membrane	Dispase II treatment/pipette	Temperature stimulus/Suprathel membrane	Dispase II treatment/pipette	
Viability of seeded cells (%)	98 ± 1.5	98 ± 1.5	98 ± 1.5	98 ± 1.5	p ≥ 0.05
Detachment yield (%)	98 ± 2	50 ± 2.5	92 ± 1	37 ± 4	p = 0.016
Re-adhesion yield (%)	72 ± 2.5	58 ± 2.2	88 ± 9	24 ± 1.1	p = 0.003
Transfer yield^a^ (%)	71 ± 3.3	29 ± 1.2	81 ± 2	9 ± 4.7	p < 0.001
Viability of re-adhered cells (%)	96 ± 2.4	80 ± 1.1	98 ± 1.6	77 ± 3.4	p = 0.008

^a^Calculated as: detachment yield × re-adhesion yield [%] values are given as mean ± standard deviation

### Cell culture and detachment from the UpCell dishes

Fibroblasts (250,000 cells per 1 cm^2^) were seeded onto UpCell dishes and onto TCPS for comparison. The cell sheet separation was performed after 24 h of fibroblast culture at 37 °C as by that time, full cell coverage of the culture dishes was obtained on both the UpCell dishes and the TCPS.

**Fig. 3:**
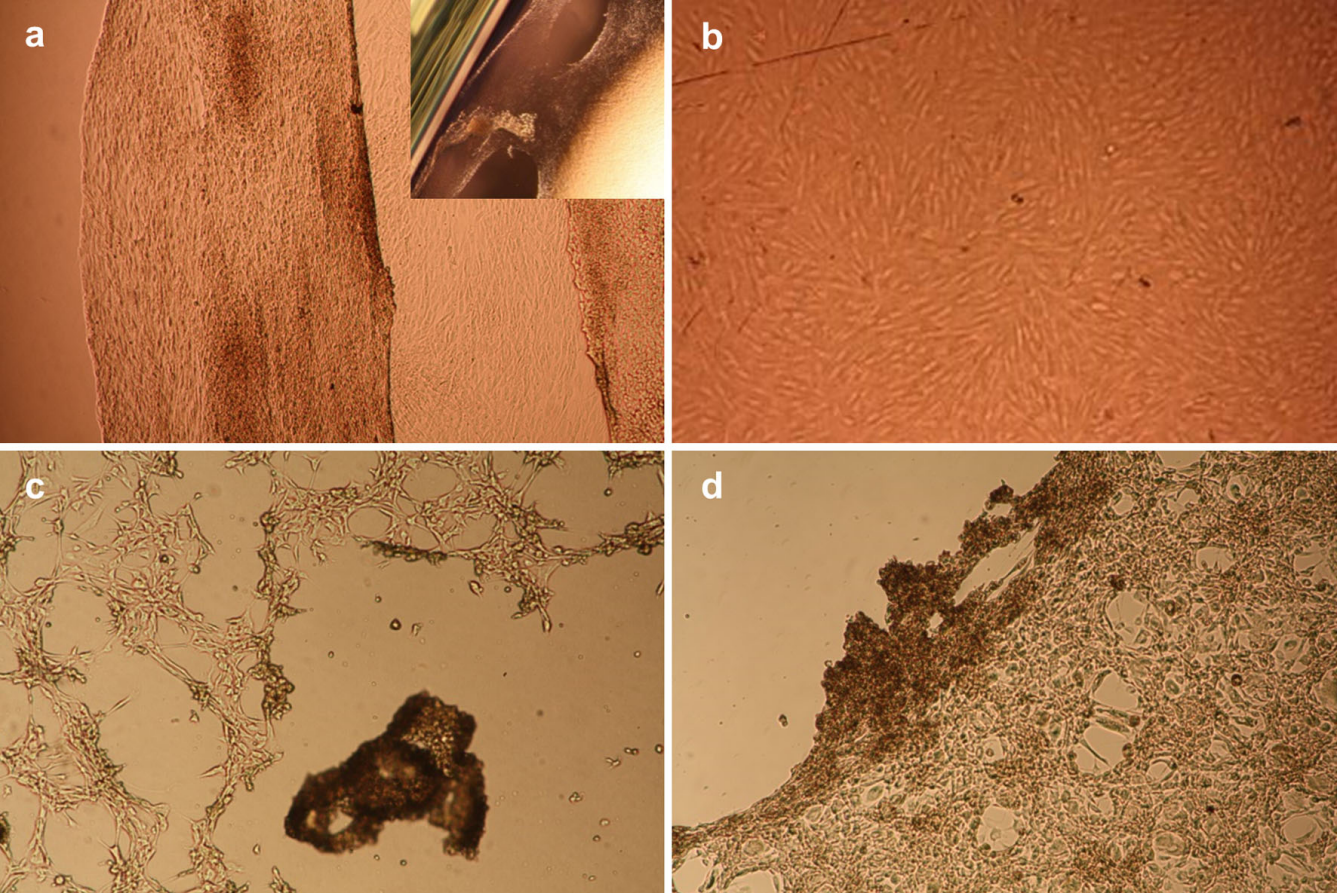
Fibroblast detachment at 20 °C from the **a** UpCell dishes and **b** TCPS dishes and at 37 °C with the use of dispase II from **c** UpCell dishes and **d** TCPS dishes (250,000 cells per 1 cm^2^, 24 h of culture, 100 % confluence)

The cell separation from the UpCell dishes was performed by reducing the temperature as described in the manufacturer’s guidelines. Fibroblasts completely separated from the UpCell dishes after 40 min of incubation at 20 °C. It was possible to obtain the entire cell sheet, but it was rolled up. The cell sheet was slightly perforated on the sides (Fig. 3a). In the case of the TCPS dish, no temperature-induced cell separation was observed (Fig. 3b). The yield of temperature detachment was 88 % for UpCell and 0 % for TCPS. As a control for cell sheet detachment, cells sheets were detached from UpCell and TCPS dishes using dispase II at 37 °C. Cell sheet detachment using dispase II (Fig. 3c, d) disturbed the cell sheet integrity (damage of cell–cell junction proteins), and the detachment had to be supported by water flushing. The yield of dispase II detachment was 50 % for UpCell and 95 % for TCPS.

**Fig. 4:**
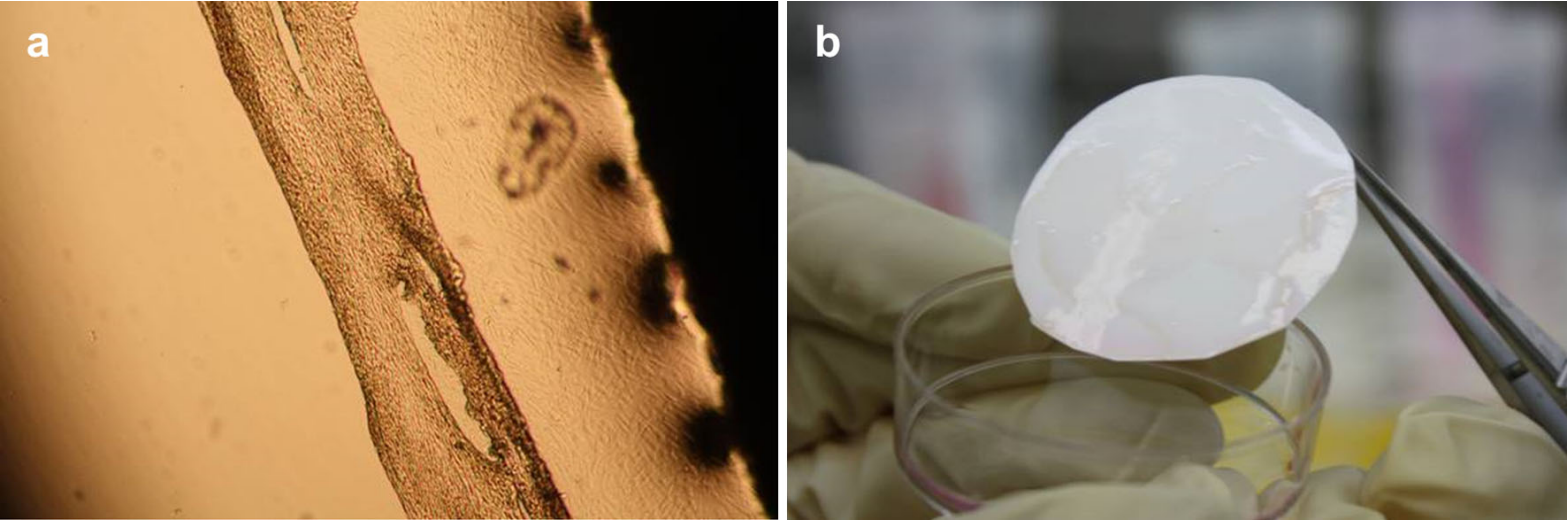
Cell sheet separation from the UpCell dish with the use of an Immobilon-P membrane: **a** a sheet of fibroblasts during separation from the dish surface and **b** a view of the UpCell dish and the transfer membrane with the cell sheet

Due to the potential applications of detached fibroblast sheets, e.g., in burn/wound treatment, we performed an experiment to relocate/transfer a full-sized sheet of fibroblasts onto another dish. A single-layer fibroblast sheet is quite fragile and rolls up when it is picked up from the medium, e.g., with forceps or a pipette. This necessitates the use of a transfer tool. According to the manufacturer’s indications, an Immobilon-P membrane was used as a transfer tool [22, 23]. After reaching full confluence, the layer of cells was covered with the membrane. Cell separation was then performed in accordance with the manufacturer’s guidelines (Fig. 4a). However, our attempts at performing such a transfer were not satisfying. Only part of the cell sheet adhered to the membrane and was transferred successfully, while a majority of the cells remained on the culture dish (Fig. 4b). The detachment yield of the cells with Immobilon-P was 65 %.

Our results showed that the Immobilon-P membrane was not efficient for relocating the fibroblast sheets. Moreover, this membrane is mainly used in scientific research (e.g., in molecular biology for western blotting [24, 25]), and it has not been accepted for clinical use.

Due to these problems with the transfer of the fibroblast sheets from the UpCell dishes, we searched for a membrane that would be appropriate for clinical use. The SUPRATHEL membrane, which is a new biocompatible and biodegradable poly(lactic acid) membrane, has properties that are similar to those of the natural epidermis. It is routinely used as a temporary skin substitute for partial thickness burns. The membrane is elastic, permeable to water vapor and impermeable to bacteria. The elasticity of the SUPRATHEL membrane allows the placement of the dressing on all types of body areas, providing a close adhesion to the damaged skin [26–28] and increasing the comfort of the patient. All of SUPRATHEL’s advantages make this membrane the leading candidate for our investigations.

**Fig. 5:**
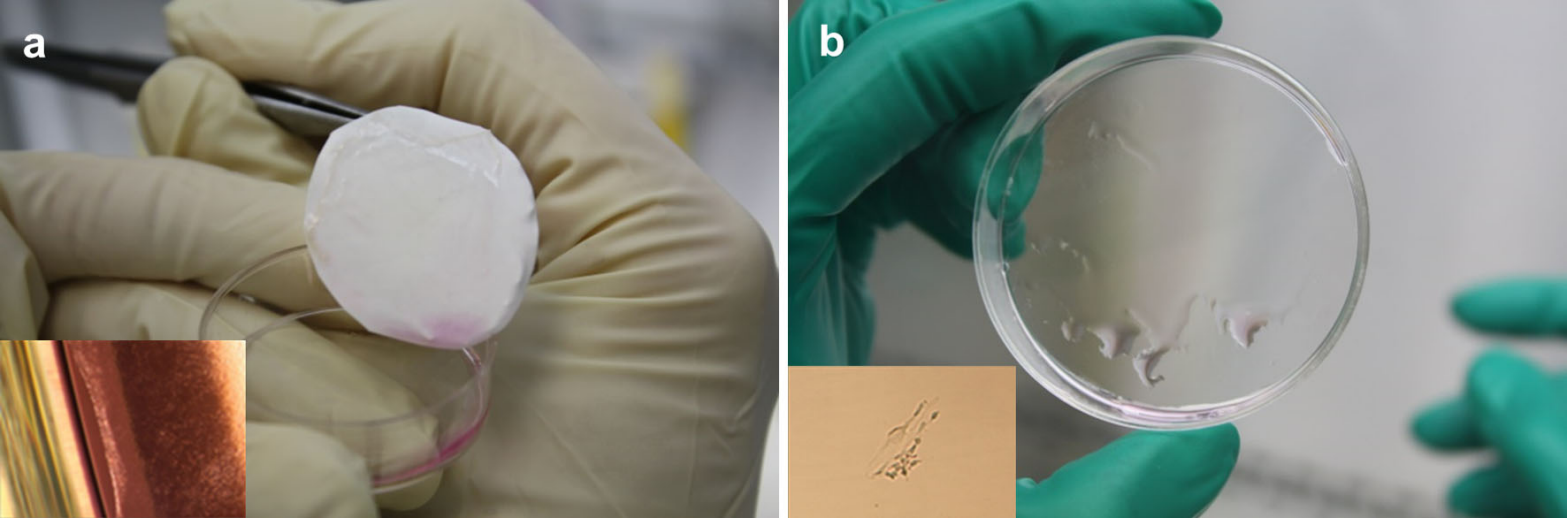
Cell sheet separation from the UpCell dish with the use of a SUPRATHEL membrane: **a** a sheet of fibroblasts on the SUPRATHEL membrane and **b** a view of the UpCell dish after removing the fibroblasts sheet with transfer membrane

A SUPRATHEL membrane was used to transfer the fibroblast sheets that were cultured on the UpCell dishes. The cell separation was performed at a reduced temperature (20°C). After the fibroblast sheet adhered to the SUPRATHEL membrane (40 min) (Fig. 5a), it was possible to transfer it to a new dish (TCPS). Small pieces of ragged cell sheets were observed only at the edge of the UpCell dish (Fig. 5b). The detachment yield of the cell sheet with SUPRATHEL was 98 %.

**Fig. 6:**
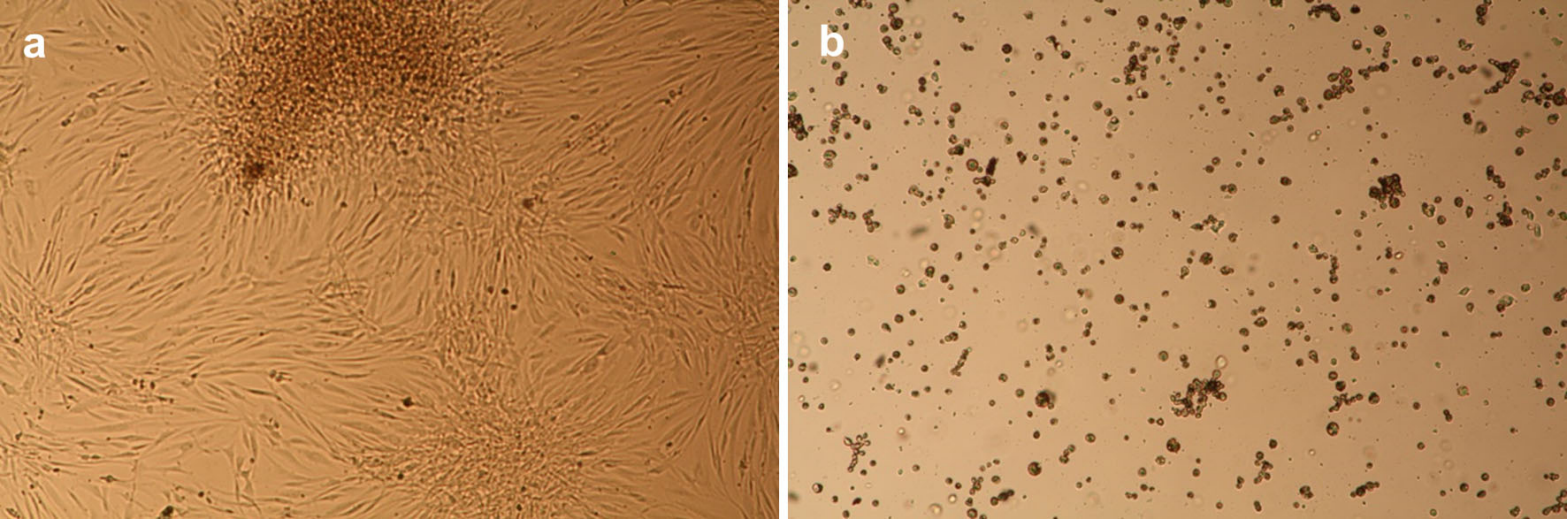
Photos of the re-adhered cells **a** transferred after temperature detachment at 20 °C from UpCell with the use of a SUPRATHEL membrane or **b** detached using dispase II at 37 °C and transferred by pipette (24 h after transfer)

In the next step, the cells’ ability to re-adhere to the new dish and the cell survival rate were investigated. The experiment was carried out for cell sheets cultured on UpCell and detached using dispase II (transferred by pipette) or by temperature reduction (transferred by SUPRATHEL membrane). The fibroblast sheets were transferred to the TCPS dish and covered with culture medium. After 24 h of re-incubation at 37 °C on the new surface, the SUPRATHEL membrane, if used, was removed. After 24 h of re-incubation (Fig. 6), the re-adhesion yield for cells detached using dispase II and transferred by pipette was 58 %, and the yield was 72 % for these detached by temperature and transferred by SUPRATHEL membrane.

The viability of the seeded cells was 98 %. The survival rate of the re-adhered cells was 96 % for cells detached by temperature stimulus and transferred by SUPRATHEL membrane, whereas the viability of cells cultured on UpCell, detached using dispase II at 37 °C and transferred by pipette was 80 %.

### Cell culture and detachment from thermoresponsive P(TEGMA-EE) dishes

**Fig. 7:**
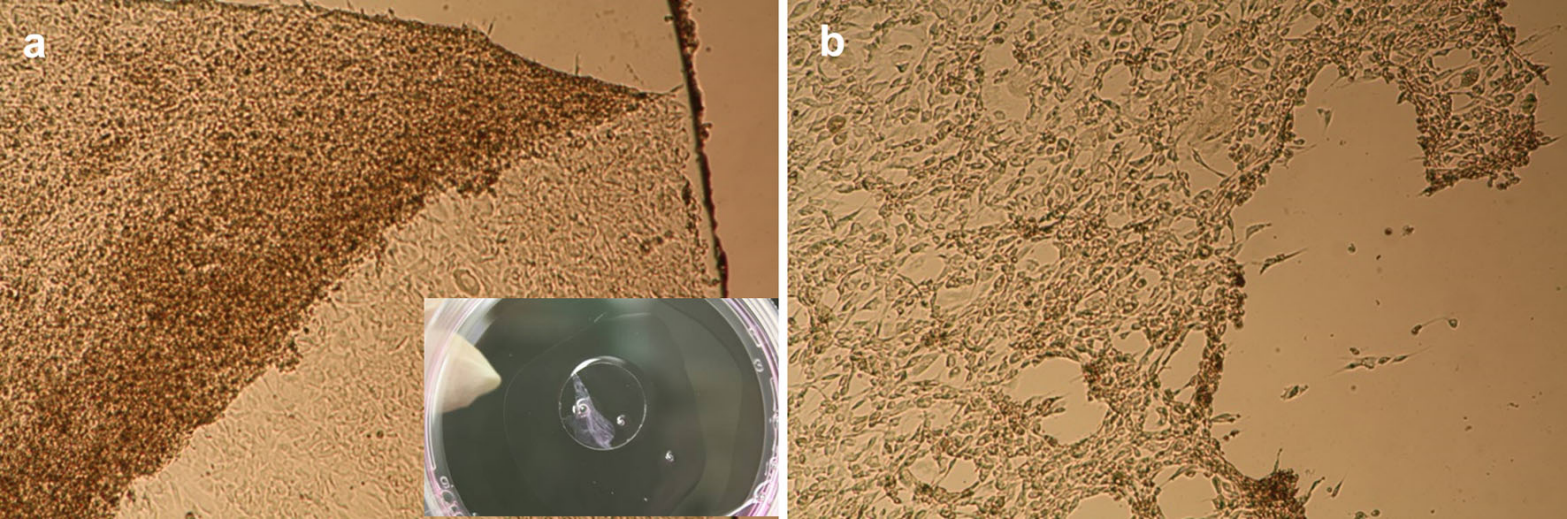
Fibroblast sheet detachment from the P(TEGMA-EE)-coated dishes at **a** 17.5 °C by temperature stimulus and **b** 37 °C with the use of dispase II (250,000 cells per 1 cm^2^, 24 h of culture, 100 % confluence)

Thermoresponsive P(TEGMA-EE) dishes were seeded with 250,000 fibroblasts per 1 cm^2^. After 24 h of cell culture, a full-sized sheet of cells was obtained. The optimal temperature for whole cell sheet detachment of 17.5 °C was determined previously [21]. Incubation of the P(TEGMA-EE)-coated dishes at that temperature produced an intact, whole sheet of cells within 40 min (Fig. 7a). The yield for temperature detachment from P(TEGMA-EE)-coated dishes was 96 %. The use of dispase II for detaching a cell sheet from P(TEGMA-EE) at 37 °C (Fig. 7b) led to 37 % cell detachment. The results are similar to those for UpCell (Fig. 3), which were described in the previous section.

**Fig. 8:**
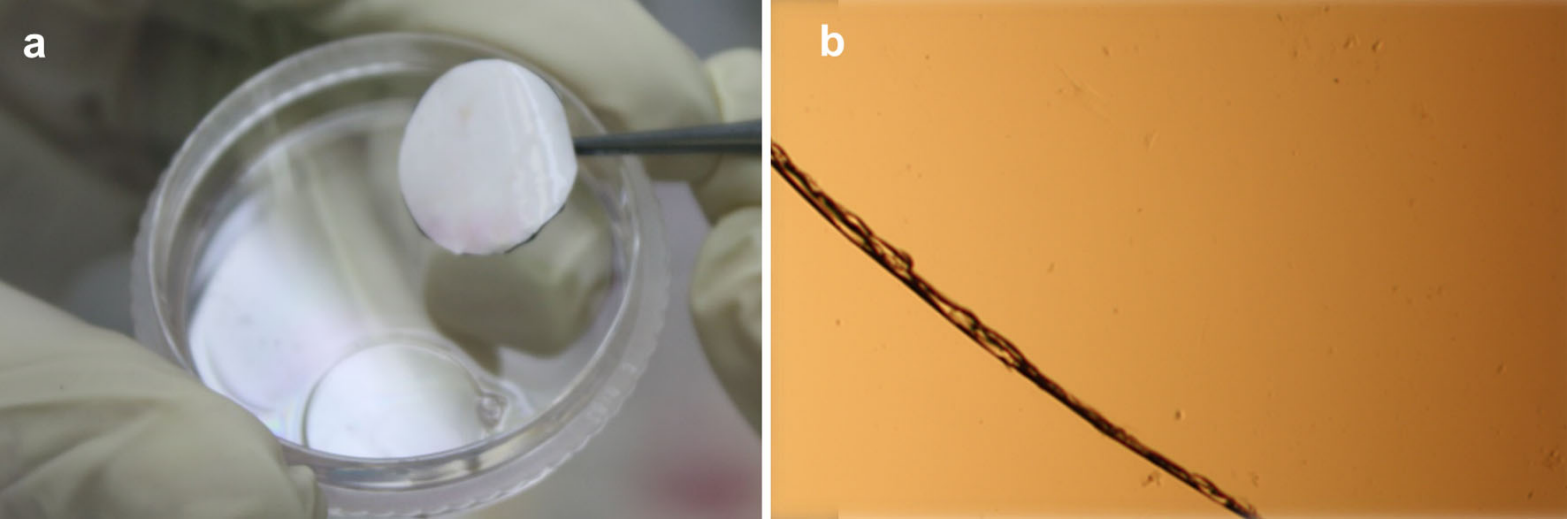
Cell sheet separation from the P(TEGMA-EE)-coated dish with the use of a SUPRATHEL membrane: **a** a sheet of fibroblasts on the SUPRATHEL membrane and **b** a view of the P(TEGMA-EE)-coated dish after removing the fibroblast sheet with a transfer membrane

Attempts to relocate the cell sheets grown on P(TEGMA-EE) dishes with SUPRATHEL membranes were performed due to the satisfactory results obtained for the transfer of the fibroblast monolayer from the UpCell dishes. Cells cultured to full confluence were covered with a SUPRATHEL membrane. Forty minutes after lowering the temperature to 17.5 °C, the SUPRATHEL membrane with the adhered cell sheet was moved from the P(TEGMA-EE)-coated dish (Fig. 8a) to a new TCPS dish. Very few cells remained on the P(TEGMA-EE)-coated dishes after membrane transfer (Fig. 8b). The detachment yield of the cell sheet with SUPRATHEL was 92 %.

**Fig. 9:**
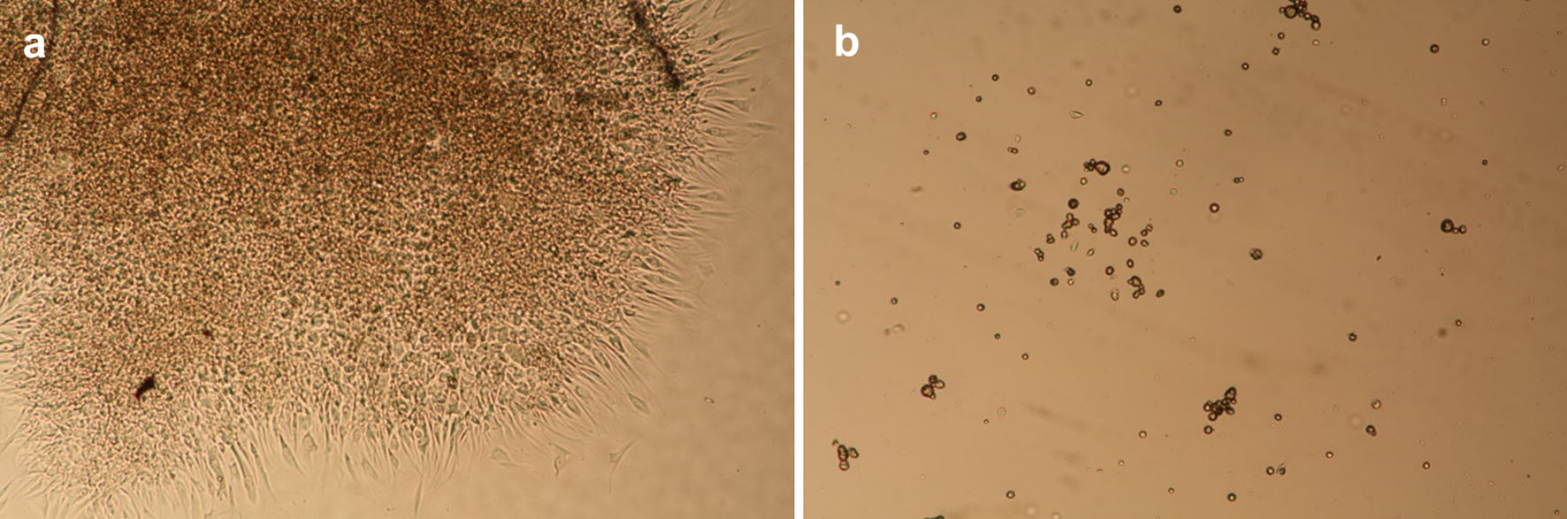
Photos of re-adhered cells **a** transferred after temperature detachment at 17.5 °C from P(TEGMA-EE)-coated dishes with the use of a SUPRATHEL membrane and **b** detached using dispase II at 37 °C and transferred by pipette (24 h after transfer)

In the next step, the cells’ ability to re-adhere to the new dish and the cell survival rate were investigated for cell sheets cultured on P(TEGMA-EE)-coated dishes and detached using dispase II (transferred by pipette) or by temperature reduction (transferred by SUPRATHEL membrane). The procedure was carried out similarly to that for UpCell dishes. After 24 h of re-incubation, the re-adhesion yield for cells detached using dispase II and transferred by pipette was 24 %, and the yield was 88 % for those detached by temperature and transferred by SUPRATHEL^®^ membrane (Table 1; Fig. 9a, b).

The characterization of cells after culture and transfer with the use of SUPRATHEL membrane from P(TEGMA-EE)-coated dishes was also performed (Table 1). The viability of the transferred fibroblasts was 98 % (the viability of the seeded cells was 98 %). For the control cells cultured on P(TEGMA-EE)-coated dishes, detached using dispase II at 37 °C and transferred by pipette, the survival rate of re-adhered cells was 77 %. The values of the tail moment olive did not reveal statistically significant difference between cells cultured and detached from P(TEGMA-EE) (TMO 4, 5 μm) and from TCPS (TMO 4, 4 μm) indicating that P(TEGMA-EE) surfaces are not genotoxic for fibroblasts.

**Fig. 10:**
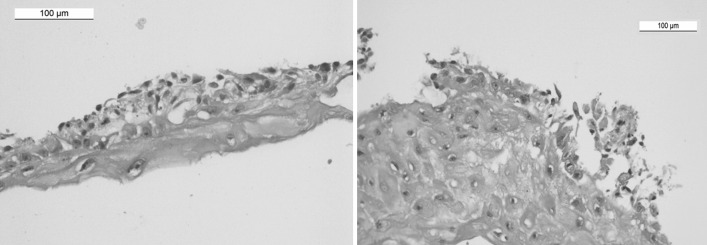
The fibroblasts sheets grown on P(TEGMA-EE) surface and spontaneously detached stained by hematoxylin-eosin

The histopathological test was done for the fibroblasts sheets grown on P(TEGMA-EE) surface and spontaneously detached. The cell sheets were fixed and stained by hematoxylin-eosin. In the histopathological specimen image (Fig. 10) the fibroblasts (dark, blue stained) placed between fibers forming the extracellular matrix can be observed.

## Discussion

At the Centre for Burns Treatment, in vitro cultured autologous keratinocytes and fibroblasts have been used for several years in clinical practice for wound healing. Cells are used as a cellular suspension in isotonic saline or in autologous platelet leukocyte-rich gel that contains growth factors [29]. Such methods enhance wound closure and enhance the formation of the epidermis. However, this procedure has some inconveniences, e.g., the need for the use of proteolytic enzymes (dispase and trypsin), which cause partial degradation of cell surface proteins and cell–cell junction proteins. This, in turn, may reduce the effectiveness of the secondary adhesion of the cells to the donor site or to the surface of the culture plates in in vitro conditions [7]. A cell suspension sprayed onto a burn wound causes a random dispersal, without the possibility of estimating the spatial distribution of the sprayed cells at different parts of the wound. At later stages of wound healing, abnormal epidermal growth is observed. Therefore, to obtain satisfactory clinical results, it is necessary to perform a few consecutive grafts [29].

The solution would be the use of supports that allow for the preparation of the sheet from epidermal and dermal cells. In our case, to obtain the cell sheet, thermoresponsive polymer-based dishes were chosen. TRPs represent a modern, high-tech group of materials that have garnered interest for many biomedical applications. TRPs have the ability to reversibly change their physical properties in response to a small, external environment temperature change [30]. These polymers are soluble in water only below a certain temperature (the so-called lower critical solution temperature (LCST) or cloud point temperature (T_CP_)), and after exceeding this temperature, they precipitate from the solution. When TRPs are covalently attached to a solid support, the properties of the resultant materials (which switch between hydrophobic and hydrophilic properties) can be altered merely by a change in the environmental temperature. Kikuchi and Okano developed the idea of cell sheet engineering with the use of supports covered with TRPs [18]. This idea involves culturing cells in the form of monolayers/sheets on supports covered with a thermoresponsive polymer and then detaching the cells by cooling the system below the phase transition temperature of the polymer. The primary advantage of applying TRPs in in vitro cell culture procedures is that there is no need to use proteolytic enzymes or mechanical separation to remove cells from the culture support.

A good example of a TRP that has been known for years is PNIPAM, which has been used to prepare surfaces for the culture and detachment of bovine aortic endothelial cells, fibroblasts, muscle cells, kidney cells, cardiac myocytes, urothelial cells, epithelial cells, hepatocytes and chondrocytes [23, 31–34]. After generating a cell sheet, a simple modification of the temperature was enough to recover cell layers from the PNIPAM surface, thereby avoiding the use of proteolytic enzymes.

Yamato’s research confirmed the validity of using PNIPAM for cell culture and its implementation in clinical practice. His experiments concerning culture and separation of corneal epithelial cells from the surface of PNIPAM showed that the thickness of the polymer layer should not be less than 20 nm [35].

In other studies, Sumide et al. proposed the use of human corneal endothelial cell sheets cultured on PNIPAM for ocular surgery and repair. Clinical scientists have been encouraged by these promising results, and further clinical research is now being conducted [36].

Our work presents an attempt to use two types of dishes covered with TRPs for fibroblast sheet formation and transfer. One of the dishes is a commercially available NUNC UpCell dish (covered with PNIPAM). The other dishes (covered with the thermoresponsive P(TEGMA-EE)) were prepared by Dworak et al. [21]. Dermal fibroblast cells derived from patients were chosen as the model cell type because during re-epithelialization these cells contribute to proper skin regeneration and facilitate wound closure.

The sheet-formed fibroblasts were detached from the surface of the UpCell dishes according to manufacturers’ guidelines at 100 % confluence when the cell sheet was formed. The lowering of the culture temperature allowed for detachment of the intact but rolled up cell sheet.

Experiments with thermoresponsive P(TEGMA-EE)-coated dishes showed promising results. In this case, cells formed a full-sized sheet of cells, which detached spontaneously only by lowering the temperature, but started to roll up afterwards.

The lowering of the culture temperature led to the detachment of the self-supporting integrated fibroblast sheet from both UpCell and P(TEGMA-EE) dishes, whereas for TCPS, the cell sheet did not detach, as expected. For comparison, an enzyme, dispase II, was used to separate cells from both types of thermoresponsive dishes. The experiment was performed at 37 °C, i.e., above the T_CP_ of the polymers forming the dish surfaces when their hydrophobicity prevents the temperature-induced detachment of cell sheets. In both cases, the dispase II detachment yield was much lower than that with temperature detachment. Moreover, as expected, the cell sheet integrity was lost; only scraps of sheet were obtained.

The cells forming a spontaneously detaching cell sheet (from UpCell and P(TEGMA-EE)-coated dishes) are flattened and closely connected. In contrast, the cells detached by dispase II, in the control experiment, were rounded and only partially connected, forming cell sheet fragments. Also, the re-adhered cells detached by trypsin were rounded. These cells were not connected and did not form sheets or cells’ aggregates.

Due to the potential application of fibroblast sheets, the solution to the problem of their transfer is very important. It was not possible to relocate the whole cell sheet with the use of the Immobilon-P membrane, which was suggested by the UpCell manufacturer. As an alternative, the SUPRATHEL membrane, which is accepted for clinical use, was used. For the first time, we showed that this membrane is an efficient tool for removing a whole, undisturbed cell sheet from both types of supports, UpCell and P(TEGMA-EE), and for transporting the cell sheet to a new plate (TCPS). The data collected in Table 1 allow for a comparison of the supports and the method of transfer of the cell sheets. The SUPRATHEL transfer yields, calculated as detachment yield × re-adhesion yield, were very high for both types of dishes compared to those for dispase II treatment. Additionally, the viability of the re-adhered cells was much higher after transfer by SUPRATHEL than by pipette. Results of histopathological examination obtained for fibroblasts sheets demonstrate that the cells are uniformly distributed between the fibers of the extracellular matrix. The genotoxicity tests performed using single cell gel electrophoresis/comet assay allowed for assessment of DNA damage. The studies showed that the thermoresponsive P(TEGMA-EE) surface are not genotoxic for fibroblasts and can be potentially applied for cell sheet preparation and their future use in clinical applications.

The use of the SUPRATHEL membrane for fibroblast sheet relocation from thermoresponsive dishes will allow for the future transfer of cells onto a patient’s wounds. SUPRATHEL, as a temporary wound dressing, can be left in place to protect the cell sheet and then removed after wound healing. Therefore, it can be expected that cell sheets transferred with SUPRATHEL may be temporarily immobilized under the membrane and protected from external factors until the cells have been incorporated into the wound. The thermoresponsive dishes might be used directly in the operating theater without any additional equipment, which would greatly simplify cell transplantation. Centre for Burns Treatment has obtained the consent of the Bioethics Committee of The Silesian Regional Medical Chamber to graft a sheet of fibroblasts cultured on P(TEGMA-EE)-coated dishes. The cell sheets were transferred by SUPRATHEL membrane to the wound bed of the patient hospitalized in Centre for Burn Treatment. Fast wound closure was achieved with very good clinical effects.

This work presents a preliminary investigation concerning the possibility of fibroblast sheet culture, non-invasive detachment from TRP-based culture dishes and effective cell sheet transfer. Considering that the proteins secreted by fibroblasts (fibronectin and collagen) promote the adhesion and proliferation of keratinocytes and that the delivery of both fibroblasts and keratinocytes onto the wound may reduce the migration of fibroblasts from subcutaneous tissue to remodel the skin tissue [20, 37], studies concerning the co-culture and transfer of sheets formed by human keratinocytes on fibroblasts are in progress.

## Conclusion

We observed that fibroblasts showed great potential for adhesion and fast growth on thermoresponsive supports. It is possible to generate the proper level of overgrowth of the polymer dishes with the fibroblast colonies over a short period of time. It is possible to detach the fibroblasts from dishes covered with the TRPs only by changing the culture temperature. However, to effectively detach the whole cell sheet, 100 % cell confluence is necessary. Both types of thermoresponsive dishes that were used as supports allowed for efficient transfer of cell sheets with the use of SUPRATHEL membranes.

The results of our experiments, including the number of cells seeded onto the polymers, the cell culture duration and transfer membrane usage, encourage more detailed research and clinical trials of in vitro cultured skin substitutes.
